# Poultry and Wild Birds as a Reservoir of CMY-2 Producing *Escherichia coli*: The First Large-Scale Study in Greece

**DOI:** 10.3390/antibiotics10030235

**Published:** 2021-02-26

**Authors:** Zoi Athanasakopoulou, Katerina Tsilipounidaki, Marina Sofia, Dimitris C. Chatzopoulos, Alexios Giannakopoulos, Ioannis Karakousis, Vassilios Giannakis, Vassiliki Spyrou, Antonia Touloudi, Maria Satra, Dimitrios Galamatis, Vassilis Diamantopoulos, Spyridoula Mpellou, Efthymia Petinaki, Charalambos Billinis

**Affiliations:** 1 Faculty of Veterinary Science, University of Thessaly, 43100 Karditsa, Greece; zathanas@uth.gr (Z.A.); msofia@uth.gr (M.S.); dchatzopoulos@uth.gr (D.C.C.); algiannak@uth.gr (A.G.); atoul@uth.gr (A.T.); 2 Faculty of Medicine, University of Thessaly, 41500 Larissa, Greece; tsilipou@uth.gr (K.T.); petinaki@uth.gr (E.P.); 3 Elanco Hellas S.A.C.I., 15231 Athens, Greece; jkarakousis@elanco.gr (I.K.); vgiannakis@elanco.gr (V.G.); 4 Faculty of Animal Science, University of Thessaly, 41110 Larissa, Greece; vasilikispyrou@uth.gr; 5 Faculty of Public and Integrated Health, University of Thessaly, 43100 Karditsa, Greece; msatra@uth.gr; 6 Hellenic Agricultural Organization DIMITRA (ELGO DIMITRA), 57001 Thessaloniki, Greece; galamatis@elog.gr; 7 Directorate of Public Health, Prefecture of Peloponnese, 22132 Tripoli, Greece; diamantopoulos@ppel.gov.gr; 8 Bioefarmoges Eleftheriou LP-Integrated Mosquito Control, 19007 Marathon, Greece; smpellou@bioefarmoges.com

**Keywords:** *Escherichia coli*, AmpC β-lactamases, antimicrobial resistance, CMY-2 type, ISE*cp1*, chickens, wild birds, livestock, Greece

## Abstract

Resistance mediated by β-lactamases is a globally spread menace. The aim of the present study was to determine the occurrence of *Escherichia coli* producing plasmid-encoded AmpC β-lactamases (pAmpC) in animals. Fecal samples from chickens (n = 159), cattle (n = 104), pigs (n = 214), and various wild bird species (n = 168), collected from different Greek regions during 2018–2020, were screened for the presence of pAmpC-encoding genes. Thirteen *E. coli* displaying resistance to third-generation cephalosporins and a positive AmpC confirmation test were detected. *bla*_CMY-2_ was the sole pAmpC gene identified in 12 chickens’ and 1 wild bird (Eurasian magpie) isolates and was in all cases linked to an upstream ISE*cp1*-like element. The isolates were classified into five different sequence types: ST131, ST117, ST155, ST429, and ST1415. Four chickens’ stains were assigned to ST131, while five chickens’ strains and the one from the Eurasian magpie belonged to ST117. Seven pAmpC isolates co-harbored genes conferring resistance to tetracyclines (*tetM, tetB, tetC, tetD*), 3 carried sulfonamide resistance genes (*sul*I and *sul*II), and 10 displayed mutations in the quinolone resistance-determining regions of *gyrA* (S83L+D87N) and *parC* (S80I+E84V). This report provides evidence of pAmpC dissemination, describing for the first time the presence of CMY-2 in chickens and wild birds from Greece.

## 1. Introduction

Antimicrobial resistance (AMR) is a globally emergent, constantly evolving threat affecting humans, animals, and the environment, thus today constituting one of the greatest One Health challenges. Bacterial resistance to cephalosporins is mainly mediated by the production of extended-spectrum β-lactamases (ESBL) and AmpC β-lactamases. AmpC enzymes confer resistance to β-lactams, with the exception of fourth-generation cephalosporins and carbapenems, and subsequently render this essential class of antibiotics ineffective [1,2]. The presence of an AmpC combined with loss of outer membrane porins can, notably, further mediate resistance to carbapenems [2,3]. Hence, although plasmid-encoded AmpC enzymes (pAmpC) are less prevalent than ESBL in most parts of the world, they may lead to resistance of a broader spectrum, while additionally being harder to detect [2].

The most common pAmpC β-lactamase reported in *Escherichia coli* (*E. coli*) isolates of both human and animal origin globally is CMY-2 [4]. The zoonotic potential of this resistance determinant is illustrated by the detection of *bla*_CMY-2_ on related plasmids and *E. coli* clones in various hosts [5,6,7]. Insertion sequences, such as ISE*cp1*, are known to play an important role in the mobilization and thus, the spread of this gene [8,9]. Among animals, poultry have been described as the most frequent *bla*_CMY-2_ carrier that can also act as an important infection source for humans, especially through meat and meat products [10,11]. On the contrary, cattle and pigs are less frequently detected to harbor this gene [12]. Alarmingly, the worldwide spread of pAmpC has additionally been evidenced in wildlife and the environment [13,14]. Wild birds play an important role as vectors of AMR and have been suggested as sentinels of circulating resistance genes within a certain geographic region [15,16]. Omnivorous, synanthropic birds are more likely to carry and disseminate resistant strains due to their vicinity to human activities and their feeding habits [17]. Despite the well documented role of animals as reservoirs and spreaders of pAmpC, their ability to directly transmit resistant bacteria to humans remains debatable [10,18].

AMR constitutes a serious threat for Greek public health. According to the surveillance report of the European Centre for Disease Prevention and Control (ECDC), Greece is classified among the countries confronting AMR the most [19], while native consumption of anti-infectives for systematic use is the highest in Europe [20]. pAmpC variants of the CMY family seem to circulate among human isolates in the country [21], while there is evidence to support that this case applies for companion animal isolates as well [22,23]. In livestock and poultry, the presence of pAmpC strains has also been ascertained [12,24]. However, there is hitherto paucity of knowledge regarding the molecular characteristics of pAmpC strains isolated from farmed and wild animals, as well as their possible relationship to human hosts.

Considering the emergence of AMR and the lack of detailed data in Greece, this study aimed to evaluate the presence of pAmpC-producing *E. coli* from poultry, cattle, pigs, and wild birds, to detect the responsible pAmpC genes and to identify the *E. coli* sequence types (ST). All pAmpC-producing *E. coli* isolates that were phenotypically resistant to antimicrobials other than β-lactams, including tetracyclines, sulfonamides, and quinolones, were further tested for the respective resistance determinants.

## 2. Results

### 2.1. Detection of pAmpC Genes in E. coli Isolates

Among the 646 animal samples, 168 were derived from wild bird species, 104 from cattle, 214 from pigs, and the remaining 159 from chickens. A total of 13 *E. coli*, 12 from chickens (12/159, 7.5%) and 1 from a Eurasian magpie (1/168, 0.6%), was found to be resistant to third-generation cephalosporins (3GC) and had a positive pAmpC-confirmation test. Molecular screening for pAmpC encoding genes revealed that all isolates carried the CMY-2 type and no other pAmpC gene type was detected in any isolate.

All strains were positive in the PCR targeting ISE*cp1 – CMY,* and sequencing analysis confirmed that *bla*_CMY-2_ genes were linked to an upstream ISE*cp1*-like element.

### 2.2. Molecular Typing

Molecular typing of the 13 isolates classified them into five different STs. ST117 *E. coli* was recovered from the wild bird as well as from five chickens. Among the remaining seven chicken strains, four were assigned to ST131 and three were identified as either ST155 or ST429 or ST1415.

### 2.3. Detection of Additional Resistance Genes

According to susceptibility testing, 12 of the 13 CMY-2-positive *E. coli* strains, including the one from the wild bird, exhibited concurrent resistance to at least three classes of antibiotics. ESBL production, by phenotypic testing, was not observed for any strain. Six strains from chickens and the one from a wild bird exhibited resistance to tetracycline (TET^R^). Out of the seven tetracycline-resistant strains, six carried *tetM,* while co-occurrence of *tetB, tetC,* and *tetD* was observed in the remaining one. Resistance to sulphonamides was expressed in two strains from chickens as well as in the one from the Eurasian magpie, which all harbored both *sul*I and *sul*II genes. Ten strains showed resistance to quinolones and fluoroquinolones (QN/FQN^R^), although none carried *qnrA, qnrB,* or *qnrS*. Sequencing analysis of the QRDRs of *gyrA* and *parC*, performed on the resistant isolates, revealed that all strains displayed a mutation of serine-83 to leucine and a mutation of aspartic acid-87 to asparagine in *gyrA*. In addition, ST131 strains also had alterations of serine-80 to isoleucine and glutamic acid-84 to valine in the QRDR of *parC*.

The antimicrobial resistance and molecular typing results of the strains are summarized in Table 1.

## 3. Discussion

In this study, pAmpC-producing *E. coli* strains were detected in 7.5% of chickens and 0.6% of wild birds, while they were not identified in cattle and pig samples. The higher frequency of pAmpC isolates among poultry, compared to other species, was in accordance with previously published data [10,12]. Their absence in cattle and pigs was expected, considering the European Union Summary Report on Antimicrobial Resistance for the years 2017 and 2018 that described low detection among fattening pigs and zero occurrence in bovine meat from Greece [12].

To the best of our knowledge, this is the first time that CMY-2 type is identified from *E. coli* isolates of farmed chickens in Greece and *bla*_CMY-2_ was the sole pAmpC gene detected, which is in agreement with previous studies [25,26,27]. Carriage was relatively low (7.5%), compared to recent reports from neighboring countries such as Turkey [28], Romania [29], and Italy [25]. Our finding may be indicative of CMY-2 type low occurrence in Greek poultry but, given the lack of previous screening studies, further investigations would be helpful to verify the aforementioned low prevalence. Considering the European prohibition of cephalosporins’ use in poultry, the emergence of ESBL/pAmpC-producing Enterobacteriaceae may be attributed to the treatment of eggs and/or one-day-old chickens in grandparent and parent flocks, along with the current management practices [30,31]. It has been shown that broilers can maintain pAmpC *E. coli* imported to the flock via one-day-old chicks or breeding animals even in the absence of selective antibiotic pressure [32,33]. This can be reflected in poultry meat, raising concern about the zoonotic capacity of pAmpC isolates.

We additionally detected a pAmpC-producing *E. coli* harbored by a Eurasian magpie (*Pica pica*) and, as far as we know, this is the first identification of CMY-2 type gene in a wild bird species from Greece. CMY-2 prevails among pAmpC *E. coli* isolates of corvids from The Czech Republic, Poland [34], Austria [16], Canada [17], and The USA [35,36], and of aquatic birds from The Netherlands [13], Spain [37], and Florida, USA [38]. We found a relatively low pAmpC carriage (0.6%) and our results are comparable with those of *Alcala* et al. [37] who reported 1.0% detection in Spain. Although higher pAmpC carriage has been published previously, varying from 3.4% in The Netherlands [13] to 26.9% in Florida [38], the low detection reported in our study could be attributed to the wide variety of the sampled wild bird species. Sampling and testing were performed, for screening purposes, not only in corvids and aquatic birds, but additionally in “low-risk” wild bird species, which are neither migratory nor omnivorous or aquatic-associated. Eurasian magpie is an omnivore and opportunistic scavenger, highly adapted to human environments and one of the most abundant corvids in Europe. Its diet and ecology, frequently interacting with humans and domestic animals, could explain the detection of a pAmpC-producing strain, as previously described for corvid populations [17]. Eurasian magpies are also known to form large communal roosts outside the breeding season, which could contribute to CMY-2 persistence and dissemination by bird-to-bird transmission during winter.

ISE*cp1* was found in the upstream region of *bla*_CMY-2_ in all our isolates. Co-existence of ISE*cp1* with ESBL/pAmpC genes in *E. coli* strains is well documented and has been associated with their efficient capture, expression, and mobilization [39,40]. Being responsible for *bla*_CMY-2_ transposition to different plasmids, ISE*cp1* probably has an important role in the dissemination of this beta-lactamase and subsequently the enhancement of its zoonotic potential [41].

MLST analysis demonstrated that the CMY-2-producing *E. coli* isolates of chickens were distributed in five different STs. Four chickens’ strains were assigned to ST131, a clone with a worldwide distribution that has contributed to the dissemination of the ubiquitous ESBL variant CTX-M-15, as well as other resistance genes [42,43]. This finding highlights the potential of acquired AmpC enzymes to arise as an important zoonotic issue. Further supporting this claim, we also detected *bla*_CMY-2_ type in a chicken *E. coli* ST155, a clone commonly reported in poultry but additionally significant for public health [44,45]. On the contrary, ST429 that was detected to express CMY-2, is a predominant avian pathogenic lineage, related only to incidental human infections [46,47]. In Greece, CMY-2-producing *E. coli* ST429 has previously been isolated from a healthy household dog [23], which could imply inter-species circulation of the clone in the country. The CMY-2 type-producing *E. coli* isolated from the Eurasian magpie (*Pica pica*) belonged to ST117, previously reported in corvids both in Europe and in Canada [17,34]. Five chickens’ isolates were also assigned to this clinically important multiresistant ST, suggesting possible strain transmission among different animal hosts in the country. Detection of ST117 in poultry and a wild bird raises concern, given its frequent association to hospital-based and community-acquired human infections worldwide [48,49,50]. Finally, an *E. coli* of chicken origin was classified as ST1415, a rather rare ST that, to our knowledge, has not been previously related to CMY-2.

Tetracycline resistance genes were identified in 6 out of the 12 CMY-2-producing poultry isolates, as well as in the Eurasian magpie isolate. Five chickens’ strains carried *tetM*, while *tetB*, *tetC,* and *tetD* were detected in the remaining one. The high frequency of tetracycline resistance among chicken pAmpC-producing isolates probably depicts the widespread use of this antibiotic in poultry husbandry all over the world [51]. Co-occurrence of *bla*_CMY-2_ and *tet* genes has formerly been reported in *E. coli* isolates from chicken carcasses in South Brazil [41], retail chicken meat in Canada [52], as well as in avian pathogenic *E. coli* from septicemic broilers in Egypt [53]. Additionally, the Eurasian magpie CMY-2 type-positive isolate displayed tetracycline resistance mediated by *tetM* and our finding complies with *Sen* et al. [35], who detected co-occurrence of *tetM* and *bla*_CMY-2_ in crow isolates.

Resistance to sulfonamides was detected in three strains, two from chickens and the one from the Eurasian magpie, which all harbored *sul*I and *sul*II sulfonamide resistance genes. In the past, sulfonamides were extensively used in traditional poultry production systems in order to achieve higher population densities and increased production. Overconsumption of this antimicrobial class resulted in the development of high resistance rates, reducing significantly its role in the poultry production nowadays [54,55]. As far as the Eurasian magpie isolate is concerned, resistance against chemically synthesized antibiotic classes such as sulphonamides has been reported in wild fauna, even though these antimicrobials are not expected to be widespread in the environment [56]. Co-occurrence of ESBL/pAmpC and sulfonamide resistance determinants on the same plasmid could probably explain the latter’s detection in the wild bird isolate [57].

Quinolone resistance was also reported in CMY-2 *E. coli* strains from nine chickens and the Eurasian magpie. Mutations were responsible for the QN/FQN^R^ phenotype and all isolates possessed the same amino acid substitution pattern in *gyrA* gene. ST131 *E. coli* possessed the S83L + D87N in *gyrA* combined with S80I + E84V in *parC*. Notably, the same mutations have been found in a collection of ST131 *E. coli* isolated from humans in Central Greece [58]. That study suggested that fluoroquinolone resistance in humans could be related to the use of these antimicrobials in the veterinary practice and the poultry production of the area. Our results verify that this specific substitutional pattern exists in *E. coli* strains of poultry origin. However, no isolate in our study co-harbored *bla*_CMY-2_ and plasmid mediated quinolone resistance (PMQR) genes, as has previously been described for ESBL/pAmpC-producing *E. coli* of poultry and wild bird origin [13,59,60].

## 4. Materials and Methods

### 4.1. Sample Collection

During 2018–2020, a total of 646 non duplicated fecal samples of clinically healthy animals were collected from different regions of Greece. In particular, 159 stool samples were collected from chickens, 104 from cattle, 214 from pigs, and 168 from thirty different wild bird species (Table 2). Samples were obtained by inserting a sterile cotton swab (Transwab^®^ Amies, UK) into the rectum or the cloaca and gently rotating the tip against the mucosa.

Regarding sampling of different wild bird species, Larsen and Australian type traps as well as modified bird catching nets were used, located in a variety of habitats. The sampling site of each wild bird was recorded using handheld Global Positioning System (GPS) units. All wild birds were released immediately following sampling, according to the prerequisites of the Greek Legislation.

Swabs were transported under refrigeration and laboratory analysis was initiated 24–48 h from the samples’ collection day.

### 4.2. Isolation, Identification and Antimicrobial Susceptibility Testing of pAmpC-producing E. coli

For the isolation of pAmpC-producing Enterobacterales, swabs were directly streaked on ESBL selective media (CHROMID^®^ ESBL, BioMérieux, Marcy l’Etoile, France) (a medium able to detect both ESBLs and high-level expressed AmpC cephalosporinases) and then the plates were incubated aerobically at 37 °C for 48 h in order to increase sensitivity [61]. Each morphologically different pink colony, corresponding to *E. coli* grown on the plates, was sub-cultured on MacConkey agar. Identification of the isolated bacteria and antimicrobial susceptibility testing were carried out using the automated Vitek-2 system (BioMérieux, Marcy l’Etoile, France), according to the manufacturer’s instructions. The antimicrobial agents tested, using the AST-GN96 card, were ampicillin, amoxicillin/clavulanic acid, ticarcillin/clavulanic acid, cefalexin, cefalotin, cefoperazone, ceftiofur, cefquinome, imipenem, gentamicin, neomycin, flumequine, enrofloxacin, marbofloxacin, tetracycline, florfenicol, polymyxin B, and trimethoprim/sulfamethoxazole. Interpretation of the antimicrobial susceptibility testing was performed automatically by the Vitek-2 software (BioMérieux, system version 8.02). Susceptibility to piperacillin/tazobactam, cefixime, cefotaxime, ceftazidime, and ceftriaxone was also tested by Etest, according to EUCAST guidelines [62].

All *E. coli* isolates that were resistant to 3GC were further tested for phenotypic AmpC production using Etest strips containing cefotetan and cefotetan plus cloxacillin (Liofilchem). Isolates that had a ratio cefotetan/cefotetan + cloxacillin ≥8 were selected for molecular detection of AmpC genes and molecular typing. Additionally, these isolates were phenotypically screened for ESBL production using Etest strips containing cefotaxime +/- clavulanic acid and Ceftazidime +/- clavulanic acid (Liofilchem). An MIC ratio ≥8 or the presence of a deformed ellipse were considered indicative of ESBL production.

### 4.3. DNA Extraction of the AmpC-Producing E.coli

Bacterial DNA was extracted from overnight cultures of the selected isolates using the PureLink^TM^ Genomic DNA Mini Kit (Invitrogen, Darmstadt, Germany), according to the manufacturer’s instructions for Gram-negative bacteria.

### 4.4. Molecular Confirmation of PAmpC Production and Screening of Insertion Sequence

In all isolates, simplex PCRs were performed for amplification of genes for the most common types of plasmid mediated AmpC β-lactamases using the primers described by Pérez-Pérez and Hanson [63] (Table 3). Post-amplification products were visualized on 2% agarose gel electrophoresis. The PCR products were purified and were analyzed by sequencing (3730xl DNA Analyzer, Applied Biosystems).

The presence of ISE*cp1* insertion element upstream of the *bla*_CMY-2_ was investigated by PCR, using a forward primer targeting the ISE*cp1* element and a reverse primer targeting the *bla*_CMY_, as described previously [41] (Table 3).

### 4.5. Molecular Typing of Isolates

Molecular typing of isolates was based on Multilocus Sequence Typing (MLST) in which amplification of seven gene loci (*adk, fumC, gyrB, icd, mdh, purA, recA*) was performed by PCR (Table 3). PCR products were purified using PureLink^TM^ PCR Purification Kit (Thermo Fisher Scientific), according to the manufacturer’s instructions. Purified products were sequenced (3730xl DNA Analyzer, Applied Biosystems) and analysis of the alleles was conducted using an online available database (https://pubmlst.org/bigsdb?db=pubmlst_ecoli_achtman_seqdef) (accessed date: 5 February 2021).

### 4.6. Molecular Detection of Additional Resistance Genes

Strains in which the presence of a pAmpC gene was confirmed and were phenotypically resistant to tetracyclines, sulfonamides, and/or quinolones were additionally tested for the respective resistance genes. In detail, genes conferring resistance to tetracycline (*tetA*, *tetB, tetC, tetD, tetM*), to sulfonamides (*sul*I, *sul*II), and the PMQR determinants (*qnrA, qnrB, qnrS*) were investigated by PCR. Quinolone-resistant isolates were also screened for mutations in the quinolone resistance-determining regions (QRDRs) of *gyrA* and *parC* by PCR and sequencing of the amplicons was performed (3730xl DNA Analyzer, Applied Biosystems) (Table 3).

## 5. Conclusions

In this study, we investigated, for the first time, the occurrence of pAmpC-producing *E. coli* from various hosts in Greece. Chicken and wild bird strains harbored *bla*_CMY-2_ type in a low prevalence, while pAmpC were not detected in cattle and pigs. ST117 and ST131 were the predominant circulating CMY-2 *E. coli* clones. Tetracycline, sulfonamide, and quinolone resistance were also identified in the CMY-2 strains, revealing the presence of *tet* genes, *sul* genes, and of mutations in the QRDRs, respectively.

## Figures and Tables

**Table 1:** Characteristics of the plasmid-encoded AmpC β-lactamase (pAmpC)-producing *E. coli* isolates.

Isolate	Host	Sequence Type	Resistance Profile		
			Phenotype	Resistance Determinants	Mutations (*gyrA*/*parC*)
C46	Chicken	ST429	AMP, AMC, TZP, CEX, CF, CEF, CFIX, CTX, CAZ, CTRX	*bla*_CMY-2_	-
C70	Chicken	ST131	AMP, AMC, TZP, CEX, CF, CEF, CFIX, CTX, CAZ, CTRX, FLU	*bla*_CMY-2_	S83L+D87N/S80I+E84V
C79	Chicken	ST131	AMP, AMC, TZP, CEX, CF, CEF, CFIX, CTX, CAZ, CTRX, FLU	*bla*_CMY-2_	S83L+D87N/S80I+E84V
C83	Chicken	ST117	AMP, AMC, TZP, CEX, CF, CFIX, CAZ, CTRX, FLU, TET, SXT	*bla*_CMY-2_, *tetM*, *sul*I, su*l*II	S83L+D87N
C88	Chicken	ST117	AMP, AMC, TZP, CEX, CF, CFIX, CAZ, CTRX, FLU, TET	*bla*_CMY-2_, *tetM*	S83L+D87N
C103	Chicken	ST117	AMP, AMC, TZP, CEX, CF, CFIX, CAZ, CTRX, FLU, TET	*bla*_CMY-2_, *tetM*	S83L+D87N
C117	Chicken	ST117	AMP, AMC, TZP, CEX, CF, CFIX, CAZ, CTRX, FLU, TET	*bla*_CMY-2_, *tetM*	S83L+D87N
C119	Chicken	ST117	AMP, AMC, TZP, CEX, CF, CFIX, CAZ, CTRX, FLU, TET, SXT	*bla*_CMY-2_, *tetM*,*sul*I, *sul*II	S83L+D87N
C136	Chicken	ST131	AMP, AMC, TZP, CEX, CF, CEF, CFIX, CTX, CAZ, CTRX, FLU	*bla*_CMY-2_	S83L+D87N/S80I+E84V
C138	Chicken	ST1415	AMP, AMC, TZP, CEX, CF, CEF, CFIX, CTX, CAZ, CTRX, TET	*bla*_CMY-2_, *tetB*, *tetC*, *tetD*	-
C147	Chicken	ST131	AMP, AMC, TZP, CEX, CF, CEF, CFIX, CTX, CAZ, CTRX, FLU	*bla*_CMY-2_	S83L+D87N/S80I+E84V
C156	Chicken	ST155	AMP, AMC, TZP, CEX, CF, CEF, CFIX, CTX, CAZ, CTRX	*bla*_CMY-2_	-
WB105	Eurasian magpie (*Pica pica*)	ST117	AMP, AMC, TZP, CEX, CF, CEF, CFIX, CTX, CAZ, CTRX, FLU, TET, SXT	*bla*_CMY-2_, *tetM*, *sul*I*, sul*II	S83L+D87N

AMP—ampicillin, AMC—amoxicillin/clavulanic acid, TZP—piperacillin/tazobactam, CEX—cefalexin, CF—cefalotin, CEF—ceftiofur, CFIX—cefixime, CTX—cefotaxime, CAZ—ceftazidime, CTRX—ceftriaxone, FLU—flumequine, TET—tetracycline, SXT—trimethoprim/sulfamethoxazole.

**Table 2:** Number of samples per wild bird species included in the study.

Common Name	Scientific Name	Number of Samples
Common blackbird	*Turdus merula*	4
Common buzzard	*Buteo buteo*	5
Common pheasant	*Phasianus colchicus*	7
Common starling	*Sturnus vulgaris*)	9
Common swift	*Apus apus*	1
Common whitethroat	*Sylvia communis*	2
Common wood pigeon	*Columba palumbus*	3
Domestic Muscovy duck	*Cairina moschata domestica*	1
Domestic goose	*Anser cygnoides domesticus*	1
Eurasian collared dove	*Streptopelia decaocto*	2
Eurasian eagle-owl	*Bubo bubo*	3
European goldfinch	*Carduelis carduelis*	6
Eurasian scops owl	*Otus scops*	1
Eurasian tree sparrow	*Passer montanus*	9
Eurasian woodcock	*Scolopax rusticola*	11
Golden pheasant	*Chrysolophus pictus*	2
Great tit	*Parus major*	5
House sparrow	*Passer domesticus*	14
Lesser kestrel	*Falco naumanni*	1
Leaf warbler	*Phylloscopus spp.*	1
Little owl	*Athene noctua*	2
Long-eared owl	*Asio otus*	2
Eurasian Magpie	*Pica pica*	52
Mallard	*Anas platyrhynchos*	3
Redwing	*Turdus iliacus*	1
Rock partridge	*Alectoris graeca*	3
Sardinian warbler	*Sylvia melanocephala*	1
Short-toed snake eagle	*Circaetus gallicus*	1
Song thrush	*Turdus philomelos*	14
Yellow-legged gull	*Larus michahellis*	1

**Table 3:** Primer sequences, amplicon sizes, and optimal annealing temperatures of each simplex PCR performed for the amplification of pAmpC and other resistance genes.

Target	Primer Sequence (5′-3′)	Amplicon Size (bp)	Annealing Temperature (°C)	Reference
**MOX** (MOX-1, MOX-2, CMY-1, CMY-8 to CMY-11)	F: GCTGCTCAAGGAGCACAGGAT	520	55	[63]
	R:CACATTGACATAGGTGTGGTGC			
**CIT** (LAT-1 to LAT-4, CMY-2 to CMY-7, BIL-1)	F: TGGCCAGAACTGACAGGCAAA	462	55	[63]
	R: TTTCTCCTGAACGTGGCTGGC			
**DHA** (DHA-1, DHA-2)	F: AACTTTCACAGGTGTGCTGGGT	405	56	[63]
	R: CCGTACGCATACTGGCTTTGC			
**ACC**	F: AACAGCCTCAGCAGCCGGTTA	346	55	[63]
	R: TTCGCCGCAATCATCCCTAGC			
**EBC** (MIR-1T ACT-1)	F: TCGGTAAAGCCGATGTTGCGG	302	58	[63]
	R: CTTCCACTGCGGCTGCCAGTT			
**FOX** (FOX-1 to FOX-5b)	F:AACATGGGGTATCAGGGAGATG	190	55	[63]
	R: CAAAGCGCGTAACCGGATTGG			
***tetA***	F: GCCTTTCCTTTGGGTTCTCT	402	55	[64]
	R: TGTCCGACAAGTTGCATGAT			
***tetB***	F: CACCACCAGCCAATAAAATT	319	52	This study
	R: TTTATTTAAAACGATGCCCA			
***tetC***	F: TCACTGGTTAACTCAGCACG	319	52	This study
	R: TCAAGTTCATTCCAACCAAT			
***tetD***	F: CTCCAATTCCCATAATTTAT	379	52	This study
	R: ATCAAAATAAAGCTAATAAC			
***tetM***	F: TTATCAACGGTTTATCAGG	398	57	This study
	R: CGTATATATGCAAGACG			
***qnrA***	F: AGAGGATTTCTCACGCCAGG	580	55	[58]
	R: CCAGGCACAGATCTTGAC			
***qnrB***	F: GGGTATGGATATTATTGATAAAG	264	55	[58]
	R: CTAATCCGGCAGCACTATTA			
***qnrS***	F: GCAAGTTCATTGAACAGGGT	428	55	[58]
	R: TCTAAACCGTCGAGTTCGGC			
***gyrA***	F: TTAATGATTGCCGCCGTCGG	648	54	[58]
	R: TACACCGGTCAACATTGAGG			
***parC***	F: GTGGTGCCGTTAAGCAAA	395	55	[58]
	R: AAACCTGTTCAGCGCCGCATT			
***sul*** **I**	F: ACG AGA TTG TGC GGT TCT TC	347	55	[64]
	R: GGT TTC CGA GAT GGT GAT TG			
***sul*** **II**	F: CCG TCT CGC TCG ACA GTT AT	506	55	[64]
	R: GTG TGT GCG GAT GAA GTC AG			
***ISEcp1 – CMY***	F- AAAAATGATTGAAAGGTGGT	546	52	[41]
	R- TTTCTCCTGAACGTGGCTGGC			

## Data Availability

Most data for this study are presented within the manuscript. The remaining data are available on request from the corresponding author. The data are not publicly available as they are part of the PhD thesis of the first author, which has not yet been examined, approved and uploaded in the official depository of PhD theses from Greek Universities.
